# 2-Azido-1-(3,6-dichloro-9*H*-fluoren-1-yl)ethanone

**DOI:** 10.1107/S1600536811036762

**Published:** 2011-09-17

**Authors:** Hoong-Kun Fun, Tze Shyang Chia, Reshma Kayarmar, Dinesha, G. K. Nagaraja

**Affiliations:** a X-ray Crystallography Unit, School of Physics, Universiti Sains Malaysia, 11800 USM, Penang, Malaysia; b Sequent Scientific Limited, Baikampady, New Mangalore, India; c Department of Chemistry, Mangalore University, Karnataka, India

## Abstract

In the title compound, C_15_H_9_Cl_2_N_3_O, an intra­molecular C—H⋯O inter­action generates an *S*(7) ring motif. The cyclo­penta-1,3-diene ring forms dihedral angles of 1.93 (6) and 2.78 (6)° with its attached benzene rings. In the crystal, mol­ecules are linked by C—H⋯N and C—H⋯O hydrogen bonds, thereby forming layers lying parallel to the *ac* plane. The crystal also features a π–π inter­action with a centroid–centroid distance of 3.5612 (6) Å.

## Related literature

For the mutagenic activity of azides, see: Sander & Muehlbour (1977 ▶); Nilan *et al.* (1973 ▶); Owais *et al.* (1983 ▶). For the preparation of 1,2,3-triazoles *via* 1,3-dipolar cyclo­addition reactions of azides with substituted acetyl­ene compounds, see: Purvisis *et al.* (1984 ▶); Patei & Smalley (1984 ▶). For a related fused-ring structure, see: Molins *et al.* (2002 ▶). For related azide structures, see: Basanagouda *et al.* (2010 ▶); Karthikeyan *et al.* (2011 ▶). For hydrogen-bond motifs, see: Bernstein *et al.* (1995 ▶). For reference bond lengths, see: Allen *et al.* (1987 ▶).
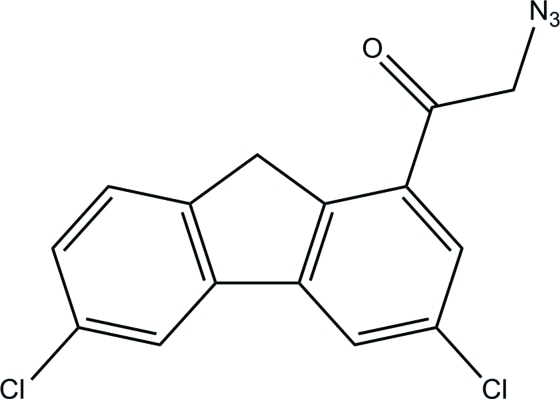


## Experimental

### 

#### Crystal data

C_15_H_9_Cl_2_N_3_O
*M* *_r_* = 318.15
Monoclinic, 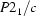

*a* = 10.7303 (1) Å
*b* = 18.7012 (3) Å
*c* = 6.8952 (1) Å
β = 98.61°
*V* = 1368.06 (3) Å^3^
*Z* = 4
Mo *K*α radiation
μ = 0.48 mm^−1^
*T* = 100 K
0.35 × 0.21 × 0.14 mm

#### Data collection

Bruker SMART APEXII CCD area-detector diffractometer
Absorption correction: multi-scan (*SADABS*; Bruker, 2009 ▶) *T*_min_ = 0.850, *T*_max_ = 0.936
15671 measured reflections
4003 independent reflections
3599 reflections with *I* > 2σ(*I*)
*R*_int_ = 0.020

#### Refinement

*R*[*F* ^2^ > 2σ(*F* ^2^)] = 0.033
*wR*(*F* ^2^) = 0.085
*S* = 1.03
4003 reflections
190 parameters
H-atom parameters constrained
Δρ_max_ = 0.37 e Å^−3^
Δρ_min_ = −0.33 e Å^−3^

### 

Data collection: *APEX2* (Bruker, 2009 ▶); cell refinement: *SAINT* (Bruker, 2009 ▶); data reduction: *SAINT*; program(s) used to solve structure: *SHELXTL* (Sheldrick, 2008 ▶); program(s) used to refine structure: *SHELXTL*; molecular graphics: *SHELXTL*; software used to prepare material for publication: *SHELXTL* and *PLATON* (Spek, 2009 ▶).

## Supplementary Material

Crystal structure: contains datablock(s) global, I. DOI: 10.1107/S1600536811036762/hb6387sup1.cif

Structure factors: contains datablock(s) I. DOI: 10.1107/S1600536811036762/hb6387Isup2.hkl

Supplementary material file. DOI: 10.1107/S1600536811036762/hb6387Isup3.cml

Additional supplementary materials:  crystallographic information; 3D view; checkCIF report

## Figures and Tables

**Table 1:** Hydrogen-bond geometry (Å, °)

*D*—H⋯*A*	*D*—H	H⋯*A*	*D*⋯*A*	*D*—H⋯*A*
C5—H5*A*⋯O1	0.95	2.32	3.0134 (14)	129
C13—H13*A*⋯N3^i^	0.99	2.59	3.4613 (16)	147
C15—H15*A*⋯O1^ii^	0.99	2.53	3.1941 (15)	125

Symmetry codes: (i) 
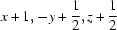
; (ii) 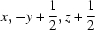.

**Fig. 1.**
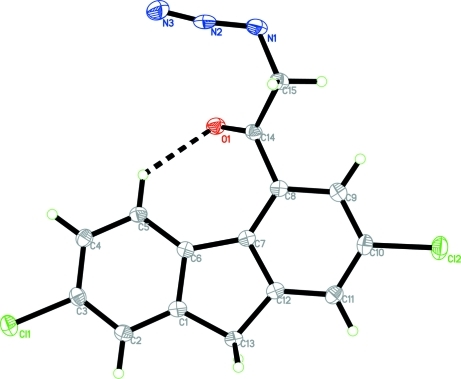
The molecular structure of the title compound with 50% probability displacement ellipsoids. The intramolecular hydrogen bond is shown by a dashed line.

**Fig. 2.**
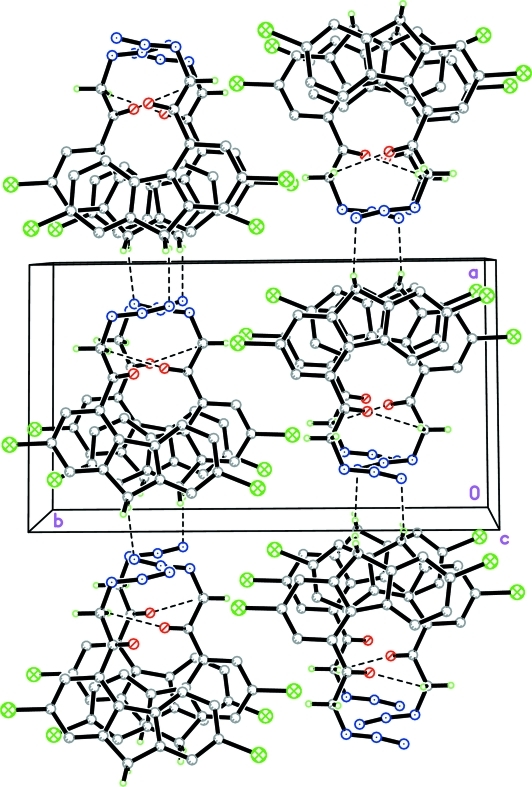
The crystal packing of the title compound. The dashed lines represent the hydrogen bonds.

## supplementary crystallographic information

### Comment

Azides are considered very important compounds due to both their industrial as well as biological applications. Azide derivatives have been used in rubber vulcanization, polymer cross linking, dyes tire cord adhesives, forming of plastics, pharmaceuticals, pesticides and herbicides. Many azide compounds show mutagenic activities (Sander & Muehlbour, 1977; Nilan *et al.*, 1973; Owais *et al.*, 1983). The chemistry of azides has thus attracted the attention of many chemists, since many of these compounds play an important role in organic chemistry. One of the more useful synthetic applications of azides is the preparation of 1,2,3-triazoles *via* 1,3-dipolar cycloaddition reactions of azides with substituted acetylene compound (Purvisis *et al.*, 1984; Patei & Smalley, 1984). The crystal structures of 4-Azidomethyl-7-methyl-2-oxo-2*H*-chromene-6-sulfonyl azide (Basanagouda *et al.*, 2010) and 2-Azidomethyl-3-methyl-1-phenylsulfonyl-1*H*-indole (Karthikeyan *et al.*, 2011) have been reported. Attributed to the above fact and with a view to obtain new and better biologically active agent, we synthesized the title compound, (I), in 60% yield.

The molecular structure of the title compound is shown in Fig. 1. The cyclopenta-1,3-diene ring (C1/C6/C7/C12/C13) makes dihedral angles of 1.93 (6) and 2.78 (6)° with its terminal benzene rings (C1–C6 & C7–C12) respectively. Bond lengths (Allen *et al.*, 1987) and angles are within normal ranges and are comparable to a related structure (Molins *et al.*, 2002). The molecular structure is stabilized by intramolecular C5—H5A···O1 hydrogen bond (Table 1) which generates an S(7) ring motif (Fig. 1; Bernstein *et al.*, 1995).

In the crystal (Fig. 2), the molecules are connected by C13—H13A···N3 and C15—H15A···O1 hydrogen bonds (Table 1) forming two-dimensional network parallel to *ac* plane.The crystal is further stabilized by π–π interactions with centroid···centroid distance, C*g*1···C*g*2 = 3.5612 (6) Å (symmetry code: *x*,1/2 - *y*,-1/2 + *z*); C*g*1 and C*g*2 are the centroids of the C1/C6/C7/C12/C13 and C1–C6 rings respectively.

### Experimental

2-Chloro-1-(3,6-dichloro-9*H*-fluoren-1-yl)ethanone (2 g, 0.0064 mole) in 5 ml DMF was cooled to 0–5 °C. Sodium azide (0.4 g, 0.0064 mole) was added lot-wise and stirred for 3 h.The precipated product was filtered off, dried and recrystallized from ethanol (1.2 g, 60%). Yellow blocks of (I) were obtained from acetone by slow evaporation.

### Refinement

All H atoms were positioned geometrically [C—H = 0.95 and 0.99 Å] and refined using a riding model with *U*_iso_(H) = 1.2 *U*_eq_(C).

### Crystal data

**Table:**

C_15_H_9_Cl_2_N_3_O	*F*(000) = 648
*M**_r_* = 318.15	*D*_x_ = 1.545 Mg m^−^^3^
Monoclinic, *P*2_1_/*c*	Mo *K*α radiation, λ = 0.71073 Å
Hall symbol: -P 2ybc	Cell parameters from 7886 reflections
*a* = 10.7303 (1) Å	θ = 2.2–30.0°
*b* = 18.7012 (3) Å	µ = 0.48 mm^−^^1^
*c* = 6.8952 (1) Å	*T* = 100 K
β = 98.61°	Block, yellow
*V* = 1368.06 (3) Å^3^	0.35 × 0.21 × 0.14 mm
*Z* = 4	

### Data collection

**Table:**

Bruker SMART APEXII CCD area-detector diffractometer	4003 independent reflections
Radiation source: fine-focus sealed tube	3599 reflections with *I* > 2σ(*I*)
graphite	*R*_int_ = 0.020
φ and ω scans	θ_max_ = 30.1°, θ_min_ = 1.9°
Absorption correction: multi-scan (*SADABS*; Bruker, 2009)	*h* = −14→14
*T*_min_ = 0.850, *T*_max_ = 0.936	*k* = −26→24
15671 measured reflections	*l* = −9→9

### Refinement

**Table:**

Refinement on *F*^2^	Primary atom site location: structure-invariant direct methods
Least-squares matrix: full	Secondary atom site location: difference Fourier map
*R*[*F*^2^ > 2σ(*F*^2^)] = 0.033	Hydrogen site location: inferred from neighbouring sites
*wR*(*F*^2^) = 0.085	H-atom parameters constrained
*S* = 1.03	*w* = 1/[σ^2^(*F*_o_^2^) + (0.0436*P*)^2^ + 0.561*P*] where *P* = (*F*_o_^2^ + 2*F*_c_^2^)/3
4003 reflections	(Δ/σ)_max_ < 0.001
190 parameters	Δρ_max_ = 0.37 e Å^−^^3^
0 restraints	Δρ_min_ = −0.33 e Å^−^^3^

### Special details

**Table:**

Experimental. The crystal was placed in the cold stream of an Oxford Cryosystems Cobra open-flow nitrogen cryostat (Cosier & Glazer, 1986) operating at 100.0 (1) K.
Geometry. All e.s.d.'s (except the e.s.d. in the dihedral angle between two l.s. planes) are estimated using the full covariance matrix. The cell e.s.d.'s are taken into account individually in the estimation of e.s.d.'s in distances, angles and torsion angles; correlations between e.s.d.'s in cell parameters are only used when they are defined by crystal symmetry. An approximate (isotropic) treatment of cell e.s.d.'s is used for estimating e.s.d.'s involving l.s. planes.
Refinement. Refinement of *F*^2^ against ALL reflections. The weighted *R*-factor *wR* and goodness of fit *S* are based on *F*^2^, conventional *R*-factors *R* are based on *F*, with *F* set to zero for negative *F*^2^. The threshold expression of *F*^2^ > σ(*F*^2^) is used only for calculating *R*-factors(gt) *etc*. and is not relevant to the choice of reflections for refinement. *R*-factors based on *F*^2^ are statistically about twice as large as those based on *F*, and *R*- factors based on ALL data will be even larger.

### Fractional atomic coordinates and isotropic or equivalent isotropic displacement parameters (Å^2^)

**Table:**

	*x*	*y*	*z*	*U*_iso_*/*U*_eq_	
Cl1	0.86335 (3)	0.015258 (16)	0.78440 (5)	0.02477 (8)	
Cl2	0.69062 (3)	0.556390 (16)	0.75596 (5)	0.02593 (9)	
O1	0.41319 (8)	0.27518 (5)	0.49870 (12)	0.02012 (18)	
N1	0.19994 (10)	0.32713 (6)	0.62768 (18)	0.0257 (2)	
N2	0.18912 (9)	0.26184 (6)	0.63263 (15)	0.0216 (2)	
N3	0.16346 (11)	0.20274 (7)	0.6281 (2)	0.0321 (3)	
C1	0.82735 (10)	0.22727 (6)	0.76076 (15)	0.0156 (2)	
C2	0.88066 (10)	0.15948 (6)	0.78230 (16)	0.0174 (2)	
H2A	0.9693	0.1533	0.8121	0.021*	
C3	0.79995 (11)	0.10118 (6)	0.75884 (16)	0.0176 (2)	
C4	0.66945 (11)	0.10914 (6)	0.71603 (16)	0.0175 (2)	
H4A	0.6166	0.0681	0.7016	0.021*	
C5	0.61693 (10)	0.17715 (6)	0.69457 (16)	0.0159 (2)	
H5A	0.5281	0.1829	0.6654	0.019*	
C6	0.69576 (10)	0.23714 (6)	0.71629 (15)	0.0143 (2)	
C7	0.67029 (10)	0.31487 (6)	0.70872 (15)	0.0143 (2)	
C8	0.55863 (10)	0.35588 (6)	0.67711 (15)	0.0155 (2)	
C9	0.56735 (11)	0.43036 (6)	0.69421 (16)	0.0181 (2)	
H9A	0.4927	0.4584	0.6765	0.022*	
C10	0.68399 (11)	0.46365 (7)	0.73686 (16)	0.0185 (2)	
C11	0.79532 (11)	0.42465 (7)	0.76498 (16)	0.0182 (2)	
H11A	0.8748	0.4478	0.7923	0.022*	
C12	0.78680 (10)	0.35082 (6)	0.75198 (15)	0.0156 (2)	
C13	0.89409 (10)	0.29828 (6)	0.78215 (16)	0.0165 (2)	
H13A	0.9442	0.3035	0.9141	0.020*	
H13B	0.9502	0.3043	0.6817	0.020*	
C14	0.43135 (10)	0.32382 (6)	0.61632 (16)	0.0164 (2)	
C15	0.32381 (11)	0.35493 (7)	0.71116 (18)	0.0207 (2)	
H15A	0.3391	0.3442	0.8533	0.025*	
H15B	0.3236	0.4076	0.6958	0.025*	

### Atomic displacement parameters (Å^2^)

**Table:**

	*U*^11^	*U*^22^	*U*^33^	*U*^12^	*U*^13^	*U*^23^
Cl1	0.02445 (15)	0.01773 (15)	0.03227 (16)	0.00395 (10)	0.00465 (11)	0.00274 (11)
Cl2	0.03214 (17)	0.01536 (15)	0.02921 (16)	−0.00146 (11)	0.00100 (12)	−0.00078 (11)
O1	0.0170 (4)	0.0224 (4)	0.0204 (4)	−0.0007 (3)	0.0008 (3)	−0.0015 (3)
N1	0.0152 (5)	0.0260 (6)	0.0360 (6)	0.0035 (4)	0.0041 (4)	0.0048 (5)
N2	0.0107 (4)	0.0299 (6)	0.0239 (5)	0.0001 (4)	0.0015 (3)	0.0012 (4)
N3	0.0203 (5)	0.0298 (7)	0.0446 (7)	−0.0036 (5)	−0.0006 (5)	0.0043 (5)
C1	0.0152 (5)	0.0198 (6)	0.0119 (5)	−0.0006 (4)	0.0026 (4)	0.0003 (4)
C2	0.0157 (5)	0.0205 (6)	0.0161 (5)	0.0016 (4)	0.0028 (4)	0.0005 (4)
C3	0.0205 (5)	0.0169 (5)	0.0158 (5)	0.0023 (4)	0.0041 (4)	0.0014 (4)
C4	0.0189 (5)	0.0174 (6)	0.0164 (5)	−0.0016 (4)	0.0035 (4)	0.0001 (4)
C5	0.0148 (5)	0.0185 (5)	0.0145 (5)	−0.0009 (4)	0.0023 (4)	0.0006 (4)
C6	0.0152 (5)	0.0175 (5)	0.0106 (4)	0.0007 (4)	0.0029 (3)	0.0005 (4)
C7	0.0150 (5)	0.0173 (5)	0.0111 (4)	−0.0007 (4)	0.0031 (3)	−0.0001 (4)
C8	0.0155 (5)	0.0187 (5)	0.0125 (4)	0.0003 (4)	0.0026 (3)	0.0001 (4)
C9	0.0202 (5)	0.0187 (6)	0.0154 (5)	0.0017 (4)	0.0027 (4)	0.0006 (4)
C10	0.0244 (6)	0.0150 (5)	0.0159 (5)	−0.0012 (4)	0.0028 (4)	−0.0006 (4)
C11	0.0195 (5)	0.0192 (6)	0.0156 (5)	−0.0030 (4)	0.0019 (4)	−0.0003 (4)
C12	0.0159 (5)	0.0187 (5)	0.0123 (4)	−0.0013 (4)	0.0027 (4)	0.0000 (4)
C13	0.0140 (4)	0.0192 (5)	0.0163 (5)	−0.0010 (4)	0.0021 (4)	−0.0007 (4)
C14	0.0150 (5)	0.0176 (5)	0.0164 (5)	0.0019 (4)	0.0018 (4)	0.0040 (4)
C15	0.0165 (5)	0.0212 (6)	0.0252 (6)	0.0022 (4)	0.0056 (4)	0.0007 (4)

### Geometric parameters (Å, °)

**Table:**

Cl1—C3	1.7436 (12)	C6—C7	1.4787 (16)
Cl2—C10	1.7400 (13)	C7—C12	1.4115 (15)
O1—C14	1.2147 (15)	C7—C8	1.4117 (15)
N1—N2	1.2275 (16)	C8—C9	1.3997 (17)
N1—C15	1.4627 (16)	C8—C14	1.4930 (15)
N2—N3	1.1382 (17)	C9—C10	1.3895 (16)
C1—C2	1.3896 (16)	C9—H9A	0.9500
C1—C6	1.4114 (15)	C10—C11	1.3882 (17)
C1—C13	1.5055 (16)	C11—C12	1.3856 (17)
C2—C3	1.3867 (17)	C11—H11A	0.9500
C2—H2A	0.9500	C12—C13	1.5042 (16)
C3—C4	1.3951 (16)	C13—H13A	0.9900
C4—C5	1.3901 (16)	C13—H13B	0.9900
C4—H4A	0.9500	C14—C15	1.5244 (16)
C5—C6	1.3995 (16)	C15—H15A	0.9900
C5—H5A	0.9500	C15—H15B	0.9900
			
N2—N1—C15	115.27 (10)	C10—C9—H9A	119.7
N3—N2—N1	171.32 (12)	C8—C9—H9A	119.7
C2—C1—C6	121.67 (11)	C11—C10—C9	121.55 (11)
C2—C1—C13	127.74 (10)	C11—C10—Cl2	119.25 (9)
C6—C1—C13	110.59 (10)	C9—C10—Cl2	119.21 (9)
C3—C2—C1	117.70 (10)	C12—C11—C10	117.86 (11)
C3—C2—H2A	121.2	C12—C11—H11A	121.1
C1—C2—H2A	121.2	C10—C11—H11A	121.1
C2—C3—C4	122.04 (11)	C11—C12—C7	122.41 (10)
C2—C3—Cl1	119.03 (9)	C11—C12—C13	126.89 (10)
C4—C3—Cl1	118.93 (9)	C7—C12—C13	110.70 (10)
C5—C4—C3	119.88 (11)	C12—C13—C1	102.69 (9)
C5—C4—H4A	120.1	C12—C13—H13A	111.2
C3—C4—H4A	120.1	C1—C13—H13A	111.2
C4—C5—C6	119.55 (10)	C12—C13—H13B	111.2
C4—C5—H5A	120.2	C1—C13—H13B	111.2
C6—C5—H5A	120.2	H13A—C13—H13B	109.1
C5—C6—C1	119.17 (11)	O1—C14—C8	122.46 (10)
C5—C6—C7	132.74 (10)	O1—C14—C15	121.08 (10)
C1—C6—C7	108.05 (10)	C8—C14—C15	116.46 (10)
C12—C7—C8	118.58 (11)	N1—C15—C14	113.40 (10)
C12—C7—C6	107.91 (9)	N1—C15—H15A	108.9
C8—C7—C6	133.44 (10)	C14—C15—H15A	108.9
C9—C8—C7	118.89 (10)	N1—C15—H15B	108.9
C9—C8—C14	118.04 (10)	C14—C15—H15B	108.9
C7—C8—C14	123.01 (10)	H15A—C15—H15B	107.7
C10—C9—C8	120.69 (11)		
			
C6—C1—C2—C3	−0.08 (16)	C14—C8—C9—C10	−175.49 (10)
C13—C1—C2—C3	178.91 (10)	C8—C9—C10—C11	−0.36 (17)
C1—C2—C3—C4	−0.31 (16)	C8—C9—C10—Cl2	179.77 (8)
C1—C2—C3—Cl1	179.91 (8)	C9—C10—C11—C12	−0.91 (16)
C2—C3—C4—C5	0.37 (17)	Cl2—C10—C11—C12	178.96 (8)
Cl1—C3—C4—C5	−179.85 (8)	C10—C11—C12—C7	0.99 (16)
C3—C4—C5—C6	−0.03 (16)	C10—C11—C12—C13	−178.30 (10)
C4—C5—C6—C1	−0.35 (15)	C8—C7—C12—C11	0.19 (16)
C4—C5—C6—C7	−177.92 (10)	C6—C7—C12—C11	−177.22 (10)
C2—C1—C6—C5	0.41 (16)	C8—C7—C12—C13	179.58 (9)
C13—C1—C6—C5	−178.74 (9)	C6—C7—C12—C13	2.18 (12)
C2—C1—C6—C7	178.53 (10)	C11—C12—C13—C1	176.92 (10)
C13—C1—C6—C7	−0.62 (12)	C7—C12—C13—C1	−2.44 (11)
C5—C6—C7—C12	176.80 (11)	C2—C1—C13—C12	−177.27 (10)
C1—C6—C7—C12	−0.96 (11)	C6—C1—C13—C12	1.82 (11)
C5—C6—C7—C8	−0.1 (2)	C9—C8—C14—O1	137.71 (12)
C1—C6—C7—C8	−177.82 (11)	C7—C8—C14—O1	−39.20 (16)
C12—C7—C8—C9	−1.45 (15)	C9—C8—C14—C15	−42.76 (14)
C6—C7—C8—C9	175.15 (11)	C7—C8—C14—C15	140.33 (11)
C12—C7—C8—C14	175.44 (10)	N2—N1—C15—C14	56.63 (15)
C6—C7—C8—C14	−7.96 (18)	O1—C14—C15—N1	−7.46 (16)
C7—C8—C9—C10	1.55 (16)	C8—C14—C15—N1	173.01 (10)

### Hydrogen-bond geometry (Å, °)

**Table:**

*D*—H···*A*	*D*—H	H···*A*	*D*···*A*	*D*—H···*A*
C5—H5A···O1	0.95	2.32	3.0134 (14)	129
C13—H13A···N3^i^	0.99	2.59	3.4613 (16)	147
C15—H15A···O1^ii^	0.99	2.53	3.1941 (15)	125

Symmetry codes: (i) *x*+1, −*y*+1/2, *z*+1/2; (ii) *x*, −*y*+1/2, *z*+1/2.
